# Evaluation of a Methylcellulose and Hyaluronic Acid Hydrogel as a Vehicle for Rectal Delivery of Biologics

**DOI:** 10.3390/pharmaceutics11030127

**Published:** 2019-03-19

**Authors:** Andreea Aprodu, Julia Mantaj, Bahijja Raimi-Abraham, Driton Vllasaliu

**Affiliations:** Faculty of Life Sciences & Medicine, School of Cancer and Pharmaceutical Sciences, King’s College London, London SE1 9NH, UK; andreea.aprodu@kcl.ac.uk (A.A.); julia.mantaj@kcl.ac.uk (J.M.); bahijja.raimi-abraham@kcl.ac.uk (B.R.-A.)

**Keywords:** biologics, biologics delivery, hyaluronic acid, hydrogels, inflammatory bowel disease, intestinal delivery, methylcellulose (MC)

## Abstract

Biologics have changed the management of Inflammatory Bowel Disease (IBD), but there are concerns regarding unexpected systemic toxicity and loss of therapeutic response following administration by injection. Local delivery of biologics directly to the inflamed mucosa via rectal enema administration addresses the problems associated with systemic administration. Hydrogels are potentially useful delivery vehicles enabling rectal administration of biologics. Here, we prepared a hydrogel system based on methylcellulose (MC) and hyaluronic acid (HA), which possesses mucosal healing properties, incorporating a model macromolecular drug, namely (fluorescently-labeled) bovine serum albumin (BSA). The BSA-loaded MCHA hydrogel showed temperature-dependent gelation (liquid-like at 20 °C and gel-like at 37 °C) and shear thinning behavior, with these being important and desirable characteristics for rectal application (enabling easy application and retention). BSA release from the MCHA system at 37 °C was linear, with 50% of the loaded drug released within 2 h. The system demonstrated acceptable toxicity towards intestinal (colon) Caco-2 epithelial cells, even at high concentrations. Importantly, application of the BSA-loaded MCHA hydrogel to polarized Caco-2 monolayers, with or without an exemplar absorption enhancer, resulted in transintestinal permeability of BSA. The study therefore indicates that the MCHA hydrogel shows potential for topical (rectal) delivery of biologics in IBD.

## 1. Introduction

Crohn’s Disease (CD) and Ulcerative Colitis (UC), two main forms of inflammatory bowel disease (IBD), are chronic, disabling and progressive diseases. Presently, conventional, small drug molecule therapy leads to symptomatic improvement but does not fully address the underlying inflammation and disease course. Biological drugs (e.g., peptides and proteins) such as infliximab (Remicade and the biosimilars Inflectra and Remsima) and adalimumab (Humira) have had a clear impact on the management of IBD. However, they currently require injection-mediated administration, which comes with important limitations, including systemic toxicity [1], loss of sustained response to therapy with time (producing symptom flares) [2], and disadvantages associated with injections such as high costs, patient discomfort and injection site injury. 

Local drug delivery to the sites of inflammation would minimize systemic drug availability, which is critical in overcoming some of the current limitations to systemic delivery of biologics in IBD. Enemas enable local drug delivery to the inflamed tissue and this method of drug delivery is routinely used in mild-to-moderate colitis. However, enema preparations also suffer from limitations such as patients not being able to retain liquid enemas for prolonged periods [3] and foam-based enemas not being suitable for access or delivery to more remote tissue areas (which can be accessible with a liquid enema) [4]. These drawbacks of enema formulations are currently being addressed, with examples including mucoadhesive hydrogels [5].

Current research efforts to improve enema formulations mainly focus on local delivery of small drug molecules. For example, a mucoadhesive hydrogel based on catechol modified-chitosan was recently shown to improve the efficacy of rectal sulfasalazine administration in a mouse model of UC, producing a lower plasma concentration of a potentially toxic drug by-product [5]. Another study reported a thermo-sensitive system, namely non-ionic surfactant copolymer based on hydrophilic polyethylene glycol and hydrophobic polypropylene glycol blocks for rectal delivery of budesonide. This thermoresponsive system (liquid at room temperature and viscous gel at body temperature), exhibited improved colonic retention compared to liquid enema [6]. 

Rectal administration could potentially achieve safe and effective local delivery of biologics, with the biotherapeutic being delivered directly to the sites of inflamed tissue, resulting in reduction in systemic exposure. Rectal delivery could also reduce the therapy costs associated with injections. The poor absorption of macromolecules across the intestinal mucosa [7,8] is noted and remains a challenge, but the issue could be addressed via the use of permeation enhancers [9,10,11] or nanotechnology approaches [7,12,13] to improving delivery of macromolecules across the intestinal mucosa. It is also possible that a defective intestinal barrier observed in IBD [14,15,16] could allow some access of biologics into the subepithelial space, although it is not presently clear whether the defective barrier could be exploited in this manner.

We recently reported a hydrogel system based on a generally recognized as safe (GRAS) amphiphile known as ascorbyl palmitate (AP), incorporating a model macromolecular drug (fluorescently-labeled dextran) [17]. The formulation of this system was inspired by a prior study [18] showing that this system displays ‘inflammation-targeting’ properties, whereby dexamethasone-incorporated AP hydrogel was delivered in a selective manner to inflammation sites as a result of charge interaction, namely negatively charged hydrogel interacting with the positively charged proteins accumulated selectively at inflammation sites [19,20]. 

In the present study, we report a simple, thermoresponsive hydrogel system based on methylcellulose (MC)—hyaluronic acid (HA) blend (MCHA) as a previously unreported candidate for rectal delivery of biologics in IBD. Bovine serum albumin (BSA, fluorescently-labeled) was incorporated into the system. The hydrogel displayed a desirable gelling behavior that is useful for rectal application (liquid-like at room temperature and gel-like at 37 °C), in addition to drug release profile, cytotoxicity and delivery to an intestinal model. The present study therefore for the first time provides evidence that the MCHA hydrogel holds potential as a safe and inexpensive system for rectal delivery of biologics in IBD.

## 2. Materials and Methods

### 2.1. Materials

Fluorescein isothiocyanate-labeled bovine serum albumin (FITC-BSA) was purchased from Sigma-Aldrich (Poole, UK). Methocel A15LV (methylcellulose, MC) was supplied by Dow Chemical Company (Midland, MI, USA). Hyaluronic acid (HA) sodium salt from Streptococcus equi was purchased from Sigma-Aldrich (Poole, UK). Transwell cell culture inserts (polycarbonate filter, 1.1 cm^2^ diameter, 0.4 μm pores) were obtained from Corning (Corning, NY, USA). Hank’s Balanced Salt Solution (HBSS), phosphate-buffered saline (PBS), trypsin, Dulbecco’s Modified Eagle Medium (DMEM), Triton X-100, and sodium dodecyl sulfate (SDS) were purchased from Sigma-Aldrich (Poole, UK). AlamarBlue^®^ cell toxicity agent was supplied by Thermo Fisher Scientific (Waltham, MA, USA).

### 2.2. Preparation of Hydrogel

MCHA hydrogel was generated by preparation of a 7.0% *w*/*v* MC solution by hot dispersion method. 4 mL HBSS was heated to 70 °C; 0.70 g MC was added to the solvent. The mixture was mixed with a magnetic stirrer for 10 min until complete powder wetting was achieved. 6 mL of HBSS was added to the slurry, followed by refrigeration at 4 °C for 24 h prior to use. As the temperature decreased, the polymer became more water-soluble, allowing complete hydration. 0.45% *w*/*v* HA solution was made by dissolving HA in HBSS with overnight stirring (using a magnetic stirrer). After preparation, the two solutions were mixed in equivalent volumes. 

For FITC-BSA-loaded MCHA hydrogels, FITC-BSA was added directly to the MCHA hydrogel and mixed manually (with a stirring rod) in order to achieve 0.2% *w*/*v* concentration and then stored in the fridge at 4 °C until further use.

### 2.3. Rheological Characterization

The rheological assessment of MCHA hydrogels was performed through a 35 mm diameter and 4° cone dimensions cone-and-plate rheometer (Physica MCR 51, Anton Paar, Austria). The samples were placed into the gap between the cone and plate to control the temperature. Triplet experiments were performed for each sample using separately prepared hydrogel batches. The rheometer was set to either 20 or 37 °C to compare the viscosity-shear rate profile at different temperature. The range of shear rate was from approximately 1 to 100 s^−1^. Rheological profile was analyzed by plotting the viscosity-shear rate profile.

### 2.4. Drug Release from Hydrogels

For drug release studies, Transwell inserts were used as filters onto which the FITC-BSA-loaded MCHA hydrogel was applied, with HBSS present in the acceptor chamber at 37 °C. 100 μL aliquots were sampled every 5 min for the first 30 min, then every 15 min for the next 30 min, every 30 min for the second hour, followed by hourly sampling for the next three hours, with the last sampling point performed after 29 h. The sampled volume was replaced with fresh HBSS. FITC-BSA was quantified by fluorescence using a TECAN Pro200 instrument (Tecan Trading AG, Männedorf, Switzerland). 

### 2.5. Cytotoxicity Assays

*AlamarBlue*^®^*assay.* For cytotoxicity study, Caco-2 cells were seeded on 48-well plates at 10^5^ cells/well and cultured for 72 h. Prior to application of different concentrations of BSA-MCHA hydrogel, the cells were washed with HBSS. BSA-MCHA hydrogel was then applied to the cells at different concentrations, specifically 0.39%, 0.78%, 1.56%, 3.125%, 6.25%, 12.5%, 25%, 50%, 75% and 100% hydrogel in HBSS. HBSS used as the negative control and 1% (*v*/*v*) Triton X-100 as the positive control. The cells were incubated with the samples for 3 h. Cell viability was determined via 2h cell incubation with 10% (*v*/*v*) AlamarBlue^®^ reagent, followed by absorbance readings at 600 nm using a TECAN Pro200 instrument.

*LDH assay.* The LDH cytotoxicity study was conducted on 96-well cultured Caco-2 cells (seeded at 5 × 10^4^ cells/well). BSA-containing MCHA hydrogel was added to the cells in HBSS at concentrations as per the AlamarBlue® assay (previous section), with HBSS and Triton X-100 used as negative and positive controls, respectively. Cells were incubated with the samples and controls for 2 h (at 37 °C and 5% CO_2_). Following the 2h-incubation period, 50 μL of media were transferred onto a new 96-well plate to which the LDH reagent was added and the solutions incubated for 30 min. The rest of the assay procedure was conducted according to the manufacturer’s instructions. 

### 2.6. Permeability Assay

Caco-2 cells were seeded at 1 × 10^5^ cells/well on Transwell inserts and cultured for 21 days. Prior to commencing the permeability experiments, transepithelial electrical resistance (TEER) was measured to confirm cell monolayer integrity. Only cell monolayers displaying TEER > 500 Ω cm^2^ were used for permeability studies. Cell culture medium (DMEM) was then replaced with warmed HBSS (37 °C) and cells allowed to equilibrate in HBSS for 30 min. BSA-containing MCHA was applied on the apical side of cell monolayers in HBSS in order to achieve a 3.125% concentration of hydrogel; the basal compartment contained 1.5 mL of HBSS. 

In the study incorporating SDS as an exemplar absorption enhancer, BSA-loaded MCHA hydrogel was applied in combination with SDS. The final concentrations of SDS applied to the cell monolayers amounted to 0.0025% and 0.01% *w*/*v*. Following the application of the samples, cell monolayers were incubated at 37 °C, with shaking. BSA permeability was assessed over four hours, with regular sampling (100 μL every hour) from the basolateral compartment. The sampled aliquots were transferred in a black 96-well plate for quantitation of FITC-BSA by fluorescence. 

### 2.7. Statistical Analysis

Unpaired, unequal variance *t*-test (or Welch *t*-test) was used for comparisons of two group means, while one way analysis of variance (ANOVA) was employed for comparison of three or more group means. *p* value of < 0.05 was considered statistically significant. ***, ** and * indicate *p* < 0.001, *p* < 0.01 and *p* < 0.05, respectively, whereas “ns” denotes nonsignificant. Statistical analysis was conducted using GraphPad Prism^®^ Software (San Diego, CA, USA). 

## 3. Results

The MCHA hydrogel formulated in this work composed of 3.5% *w*/*v* MC and 0.225% *w*/*v* HA and incorporated 0.2% *w*/*v* FITC-BSA as a model biological drug. This system was characterized in terms of rheological properties, model biological drug release, cytotoxicity and delivery of a model biologic in an in vitro intestinal model (Caco-2 monolayers).

### 3.1. Hydrogel Preparation 

**Figure 1:**
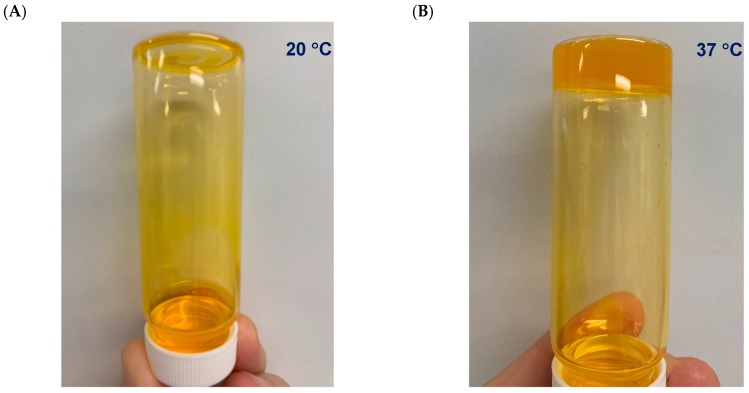
Methylcellulose (MC)-hyaluronic acid (HA) hydrogels (MCHA) incorporating fluorescein isothiocyanate-labeled bovine serum albumin (0.2% *w*/*v*) at 20 °C (**A**) and 37 °C (**B**).

In terms of the visual observations of the temperature-dependent consistency of the formulated system, Figure 1 shows the consistency of this system, which is clearly liquid-like at 20 °C (**A**) and gel-like at 37 °C (**B**).

### 3.2. Rheological Characterization

**Figure 2:**
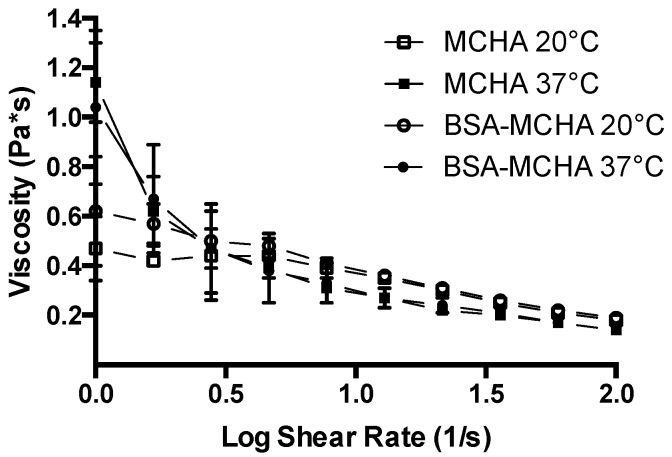
Rheological properties of the methylcellulose (MC)-hyaluronic acid (HA) hydrogels (MCHA) at 20 and 37 °C, with or without fluorescein isothiocyanate-labeled bovine serum albumin at 0.2% *w*/*v* (BSA-MCHA). Data represent means ± SD (*n* = 3).

The rheological profile of the systems formulated in this work are shown in Figure 2. MCHA hydrogels displayed a temperature-dependent rheological behavior. At 37 °C the system appeared more gel-like, while at 20 °C the consistency was more liquid-like. The rheological profile supports this characteristic as the viscosity of MCHA hydrogels at 37 °C was higher than that at 20 °C. The viscosity at both temperatures decreased with an increasing shear rate from 1 to 100 s^−1^. The viscosity versus shear rate profile was not affected by the inclusion of BSA, as evidenced from the similarity in the data for both conditions. 

### 3.3. Drug Release from Hydrogels

**Figure 3:**
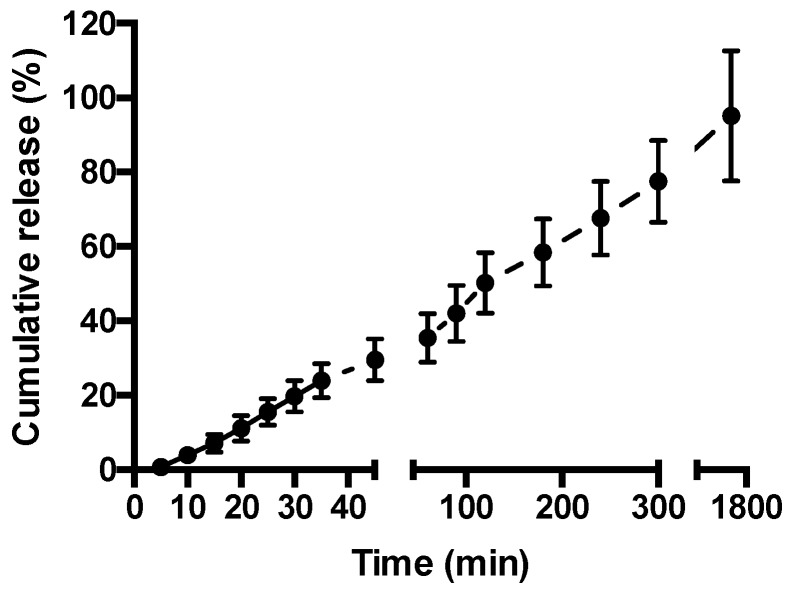
Fluorescein isothiocyanate-labeled bovine serum albumin (FITC-BSA) release from methylcellulose (MC)-hyaluronic acid (HA) hydrogel. FITC-BSA release was measured at 37 °C in Hank’s Balanced Salt Solution. Data shown as mean ± SD (*n* = 3).

Figure 3 depicts release of FITC-BSA from the hydrogel at 37 °C. A constant rate of drug release is apparent, with 50% of the drug released within 120 min, increasing to 78% at time 300 min. At the last measurement interval of 1740 min (29 h), FITC-BSA release reached 95%. 

### 3.4. Cytotoxicity Assay

**Figure 4:**
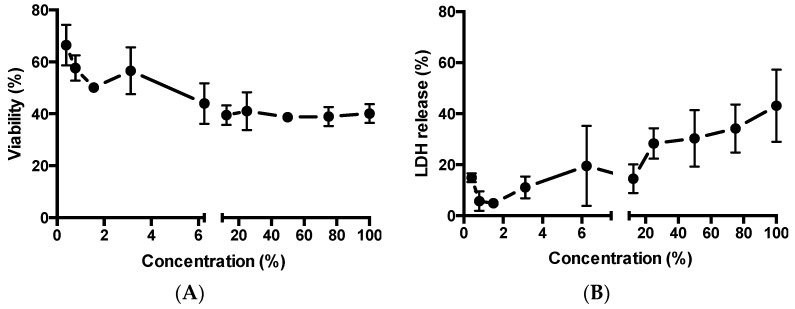
Effect of methylcellulose (MC)-hyaluronic acid (HA) (MCHA) hydrogel on toxicity to intestinal epithelial Caco-2 cells. (**A**) Effect on relative viability (*AlamarBlue*^®^ *assay*). (**B**) Effect on cellular release of lactate dehydrogenase (LDH). Data shown as the mean ± SD (*n* = 4).

Two cytotoxicity assays, based on different working mechanisms, were used to evaluate the toxicity of MCHA (containing FITC-BSA to mirror application for permeability study) towards Caco-2 cells, namely AlamarBlue^®^ (measure of cell metabolic activity) and LDH assay (indication of cell membrane damage). Figure 4A shows an overall concentration-dependent trend, with increasing concentrations of applied MCHA inducing larger effects (reduction) on cell viability. The data however shows a presently unclear plateau in reduction of cell viability at concentrations of 6.25% *v*/*v* and higher, with cell viability remaining at around 40% even following application of MCHA at high concentrations. 

Figure 4B depicts the relationship between MCHA application to Caco-2 cells at a wide concentration range and LDH release. Overall, LDH release is proportional to the concentration of applied MCHA, although there are two data points that do not fit that trend. LDH release ranged from under 5% with 1.5% MCHA to 43% with 100% MCHA.

### 3.5. Permeability Assay

**Figure 5:**
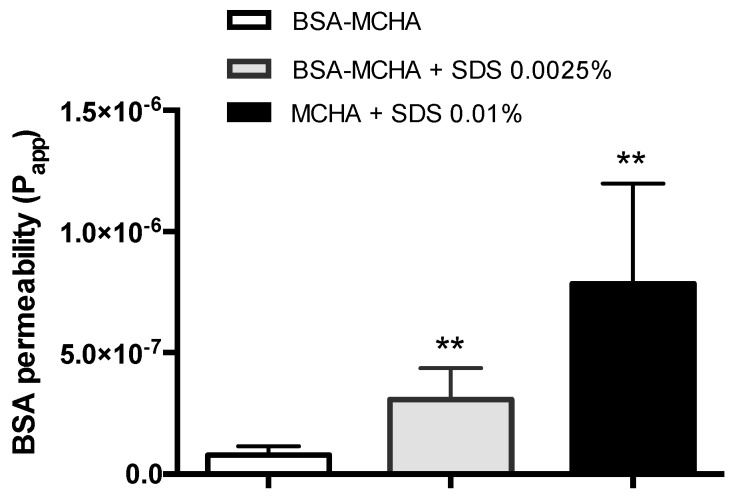
Fluorescein isothiocyanate-labeled bovine serum albumin (FITC-BSA) permeability across Caco-2 monolayers following application of FITC-BSA-containing methylcellulose (MC)-hyaluronic acid (HA) (MCHA) hydrogel, with or without sodium dodecyl sulfate at 0.0025% and 0.01% *w*/*v*. FITC-BSA permeability was quantified by fluorescence. Data shown as the mean ± SD (*n* = 4). ** indicates *p* < 0.01.

To assess the mucosal macromolecular delivery potential of the MCHA hydrogel, the system was formulated incorporating an exemplar absorption-enhancing excipient, namely SDS. The permeability of FITC-BSA following the application of SDS and FITC-BSA-containing MCHA hydrogel, tested at 37 °C over four hours, is shown in Figure 5. FITC-BSA permeability across the Caco-2 monolayers was 4-fold and 10-fold higher following its application within the system containing 0.0025% and 0.01% *w*/*v* SDS, respectively, compared to the system without SDS. 

## 4. Discussion

This study investigated the MCHA hydrogel for its potential utility as a vehicle for rectal administration of biological therapeutics. MCHA hydrogel was investigated given the biocompatibility of its components [21], as well as additional desirable properties. For example, MC exhibits thermoresponsive properties and has previously been used in small bowel enemas [22]. HA is a naturally occurring glycosaminoglycan, a polysaccharide of high molecular weight that is found in extracellular matrix, and exhibits interesting viscoelastic properties, excellent biocompatibility, biodegradability [23] and mucoadhesive properties [24]. To the best of our knowledge, these systems have not previously been investigated for their potential to deliver biologics in IBD. 

While the rectal route of drug administration may not offer a high level of patient acceptability that is associated with, for example, the oral route [25], local delivery of biologics presents an opportunity to achieve high doses at inflammation sites, whilst significantly reducing the systemic exposure (and related negative consequences) and loss of therapeutic response [26]. A large number of IBD patients can benefit from topical therapies, which potentially offer effective and more rapid treatment during acute flares [6].

Hydrogels can potentially act as vehicles for delivery of biotherapeutics to the inflamed mucosal surfaces. However, while mucoadhesive or thermo-responsive hydrogels have been studied and shown potential for rectal delivery of small drug molecules [5,6,27], such systems have not been investigated for rectal delivery of biologics. We were specifically interested in the potential utility of MCHA thermoresponsive hydrogel for local delivery of biologics given the benefits of reducing systemic exposure of such therapies, together with evidence of therapeutic potential following topical application [28].

The thermoresponsive gelation of the MCHA system at body temperature is desirable since it allows easy administration as enema in liquid-like state, followed by retention of the gel. This property is attributed to MC. At low temperatures water molecules interact with its methoxyl groups via hydrogen bonding, making MC water-soluble. Temperature increase causes destruction of these hydrogen bonds, resulting in exposure of the slightly more hydrophobic regions of MC and formation of different hydrophobic interactions (intra- and inter-molecular), leading to gel network formation [29]. Therefore, this property has the advantages of liquid enema enabling more proximal delivery, but addresses retention issues associated with liquids [6]. Additional potential advantages of the MCHA hydrogel for rectal administration in IBD include the wound-healing properties of HA [30], as demonstrated by a recent study where it promoted colonic mucosal healing in 2,4,6-trinitrobenzene sulfonic acid (TNBS)-induced colitis when administered rectally in a combination with 5-ASA (HA and 5-ASA combination was more effective than 5-ASA alone) [31]. Furthermore, HA has mucoadhesive properties [24], particularly to the injured mucosa; fluorescently-labeled HA conjugates were shown to adhere on injured colon more strongly than to normal tissue [31]. Although the mucoadhesive and inflammation-targeting properties of MCHA hydrogel were not studied in the present work, the MCHA hydrogel could show these beneficial properties which would facilitate its function as a vehicle for local delivery of biologics. MCHA hydrogels are therefore envisaged to combine the thermosensitive properties of MC with a plethora of potentially beneficial properties of HA, giving rise to therapeutic properties of the delivery vehicle itself. 

We confirm that the MCHA hydrogel possesses temperature-dependent rheological behavior, with more prominent gel-like properties at 37 °C and more liquid-like characteristics at 20 °C (Figure 1 and Figure 2). The viscosity at both 37 °C and 20 °C decreased with increasing shear rate and this profile was not affected by the inclusion of BSA, as evidenced from the similarity in the data trends observed in both scenarios. This rheological behavior is beneficial both for application of the formulation (application of shear), as well as colonic retention [6], which could also permit less frequent administration, improving patient acceptability. It should be noted that the selection of concentrations and ratio of MCHA hydrogel components was based on a previous study showing stable gel formation at 37 °C [32]. There is therefore scope for further optimization of the rheological properties of the MCHA hydrogel for rectal application by modifying the concentrations and ratio of its components.

The therapeutic potential of a drug-loaded hydrogel for local drug delivery depends on the ability of the system to release the drug cargo. We therefore examined the release of incorporated model biologic (FITC-BSA) from the hydrogel at 37 °C. MCHA hydrogel demonstrated a linear (constant) rate of drug release, with 50% of the drug released within two hours (Figure 3). The profile of FITC-BSA release from the MCHA hydrogel suggests a diffusion-mediated mechanism of release (cumulative release is linear with respect to the square root of time for the first ~77% of release; R = 0.985), which mirrors the observations by Kang et al. with erythropoietin release from this system [33].

Studies evaluating the intestinal toxicity of drug-loaded MCHA utilized assays indicative of the effect on cell metabolic activity (alamarBlue^®^) and cell membrane damage (LDH assay). A concentration-dependent trend was seen, although the application of MCHA hydrogel to intestinal epithelial Caco-2 cells at high concentrations did not induce dramatic toxicity. 

Owing to the poor permeability of biologic macromolecules across the rectal and colonic mucosa, a rectally-administered formulation intended for biologic delivery would potentially have to incorporate an absorption enhancer (or, alternatively, the biologic would have to be modified in a way that its mucosal permeability is improved). Penetration of biological therapeutics across the mucosa is necessary considering that inflammation in IBD spans the mucosal wall (i.e., it is transmural) and there is a need to target the cells underneath the epithelium (e.g., tumor necrosis factor alpha [TNFα]-producing macrophages). The MCHA hydrogel was therefore tested with co-application of an exemplar absorption enhancer, SDS. The concentration of SDS used in the studies was low, specifically up to 100 times lower (0.0025% *w*/*v*) than its critical micelle concentration (CMC) of around 0.24% *w*/*v* [34], at which point SDS becomes toxic to Caco-2 cells [35], and up to five times lower than its prior use as an absorption enhancer [36,37]. Figure 5 showcases that under these circumstances, the transintestinal delivery of a large molecule (66 kDa) is significantly enhanced. Such a delivery strategy would potentially be applicable to smaller antibody fragments compared to full sized monoclonal antibody-based therapeutics for IBD. It is noted that the safety of many absorption enhancers with chronic use, particularly in diseases where the mucosal tissue is abnormal (such as IBD) is unclear, but the study nevertheless highlights the potential of the approach. 

## 5. Conclusions

Overall, this study demonstrated that MCHA hydrogel possesses desirable properties that can potentially be exploited for its use as a vehicle for rectal delivery of biotherapeutics in IBD. These include thermoresponsiveness and shear thinning properties that should result in effective application and retention as rectal enema, combined with constant release of a model macromolecular drug. Future studies should more comprehensively evaluate this potential.
